# Age-Dependent Astroglial Vulnerability to Hypoxia and Glutamate: The Role for Erythropoietin

**DOI:** 10.1371/journal.pone.0077182

**Published:** 2013-10-04

**Authors:** Ali Lourhmati, Gayane H. Buniatian, Christina Paul, Stephan Verleysdonk, Reinhild Buecheler, Marine Buadze, Barbara Proksch, Matthias Schwab, Christoph H. Gleiter, Lusine Danielyan

**Affiliations:** 1 Department of Clinical Pharmacology, Institute of Clinical and Experimental Pharmacology and Toxicology, University Hospital of Tuebingen, Tuebingen, Germany; 2 H. Buniatyan Institute of Biochemistry, National Academy of Sciences, Yerevan, Armenia; 3 German University in Cairo, Cairo, Egypt; 4 Dr. Margarete Fischer-Bosch Institute of Clinical Pharmacology, University of Tübingen, Stuttgart, Stuttgart, Germany; National Institute of Agronomic Research, France

## Abstract

Extracellular accumulation of toxic concentrations of glutamate (Glu) is a hallmark of many neurodegenerative diseases, often accompanied by hypoxia and impaired metabolism of this neuromediator. To address the question whether the multifunctional neuroprotective action of erythropoietin (EPO) extends to the regulation of extracellular Glu-level and is age-related, young and culture-aged rat astroglial primary cells (APC) were simultaneously treated with 1mM Glu and/or human recombinant EPO under normoxic and hypoxic conditions (NC and HC). EPO increased the Glu uptake by astrocytes under both NC and especially upon HC in culture-aged APC (by 60%). Moreover, treatment with EPO up-regulated the activity of glutamine synthetase (GS), the expression of glutamate-aspartate transporter (GLAST) and the level of EPO mRNA. EPO alleviated the Glu- and hypoxia-induced LDH release from astrocytes. These protective EPO effects were concentration-dependent and they were strongly intensified with age in culture. More than a 4-fold increase in apoptosis and a 2-fold decrease in GS enzyme activity was observed in APC transfected with EPO receptor (EPOR)-siRNA. Our in vivo data show decreased expression of EPO and a strong increase of EPOR in brain homogenates of APP/PS1 mice and their wild type controls during aging. Comparison of APP/PS1 and age-matched WT control mice revealed a stronger expression of EPOR but a weaker one of EPO in the Alzheimer’s disease (AD) model mice. Here we show for the first time the direct correlation between the extent of differentiation (age) of astrocytes and the efficacy of EPO in balancing extracellular glutamate clearance and metabolism in an in-vitro model of hypoxia and Glu-induced astroglial injury. The clinical relevance of EPO and EPOR as markers of brain cells vulnerability during aging and neurodegeneration is evidenced by remarkable changes in their expression levels in a transgenic model of AD and their WT controls.

## Introduction

Glutamate (Glu), the major excitatory neurotransmitter in the mammalian central nervous system (CNS), is crucial for brain functions such as learning and memory (for review, see 1). Glu homeostasis in the brain is maintained by its well-balanced release, uptake and metabolism [2]. Increased extracellular Glu concentration is known to be a common factor in the pathologies of Alzheimer’s disease (AD), Parkinson’s disease (PD), multiple sclerosis (MS) and stroke [3]. These pathological conditions are accompanied by local hypoxia, which is believed to exacerbate disease progression (for review, see 4). Aging also leads to increased extracellular Glu levels in the CNS, which in turn cause excitotoxicity and a reduction in the number of glutamatergic synapses [5,6]. Excessive activation of Glu receptors leads to intraneuronal calcium overload, free radical generation and neuronal death [7]. Advanced age impairs the delivery of oxygen to cells and tissues, rendering neurons more susceptible to damage [4].

The essential role of astroglia in cerebral Glu homeostasis is rapid uptake of extracellular Glu, followed by its conversion into and release of glutamine (Gln) for neuronal re-uptake. This pathway constitutes the astroglial part of the Glu/Gln cycle ([8], [2]]; for review, see [9]) and is a possible target of hypoxia-caused disturbance in cerebral intercellular cooperation. Astrocytic capability for Gln synthesis from Glu depends on mitochondrial citric acid cycle activity, which, in turn, depends on oxygen tension [10]. Astrocytic Glu turnover, lactic acid accumulation and glutamine synthetase (GS) activity depend on oxygen partial pressure [11].

On the other hand astrocytes, the largest subpopulation of glial cells in the CNS, are the main producers of erythropoietin (EPO) in the brain, both under normal conditions [12] and during hypoxic/ischemic insults (for review, see 13). The neuroprotective effects of EPO are reflected by decreased Glu toxicity, generation of neuronal anti-apoptotic factors, reduction of inflammation, decreased nitric oxide-mediated injury and direct antioxidant effects [13].

Most age-related neurological diseases are accompanied by hypoxia [7] and extracellular accumulation of Glu. Glu-mediated excitotoxic neuronal damage is frequently associated with impaired handling of extracellular Glu by gliotic astrocytes [3]. In vitro studies have shown that EPO protects primary cultured neurons from Glu toxicity and against ischemia-induced neuronal death [13]. EPO significantly reduces both infarct area and volume at 24h after an ischemic insult caused by permanent middle cerebral artery occlusion [14]. The above effects of EPO may underlay its therapeutic effects in different experimental models of neurodegeneration, such as AD, PD and MS (for review, see 15), or in stroke [16].

Previously we reported that EPO increases the survival of culture-differentiated astrocytes under normoxic (NC) as well as hypoxic culture conditions (HC [17]). The aim of the present study was to investigate the cell age-dependent effect of EPO on the main cellular mechanisms mediating the function of astrocytes in cerebral Glu homeostasis and metabolism, namely Glu uptake, expression of glutamate aspartate transporter (GLAST), activity of glutamine synthetase (GS) and survival under exposure to high Glu levels and hypoxia. In addition, the involvement of EPO receptor (EPOR) in survival of astrocytes during hypoxia- and Glu-induced cell death, as well as EPO and EPOR expression levels in a transgenic model of AD (APP/PS1 mice) and their age matched wild type (WT) controls were investigated.

## Materials and Methods

### Animals

For cell culture experiments timed pregnant Wistar rats were purchased from Charles River (Sulzfeld, Germany).

For detection of EPOR in the brains of a transgenic mouse model of Alzheimer’s disease, male APP/PS1 (Strain: B6.Cg-Tg(APP695)3Dbo Tg(PSEN1dE9)S9Dbo/Mmjax) were purchased from Jackson Labs (Bar Harbour, ME). Age matched C57 BL/6 mice (Charles River) served as a WT controls.

Animals were kept under a 12 hours dark to light cycle, with food and water ad libitum. Eight and 13 month-old mice (n=5 in each group) were sacrificed under ketamine anesthesia (75mg/kg i.p., Delta Select, Pfullingen, Germany). The brains were frozen at -80°C and processed as described below.

### Ethics statement

All procedures were carried out in accordance with the guidelines for the care and use of laboratory animals of the German Animal Protection Law. The research protocol was approved by the institutional board of the animal facility of the University Hospital of Tübingen and the local governmental authorities (Regierungspräsidium Tübingen, permit number §4 Abs. 3 Az v. 13.02.09 and §4 Abs. 3 Az v. 27.02.13).

### Cell culture preparation

Astroglial primary cultures (APC) were prepared from the brains of newborn Wistar rats as described elsewhere [18]. Briefly, the cells were mechanically dissociated, centrifuged and cultured in Dulbecco’s modified Eagle’s medium (DMEM) with 4,5g/l Glucose supplemented with 3.7g/l bicarbonate, 10% fetal calf serum, 100µg/ml streptomycin sulfate, 100 units/ml penicillin G and 100 mM pyruvate (Biochrom AG, Berlin, Germany) in a humidified 10% CO_2_ atmosphere at 37°C.

For immunocytochemical studies, viable astroglial cells (3x10^6^) were seeded in culture dishes containing glass cover slips. For the cytotoxicity and viability assays, as well as the measurement of caspase-3/-7 activity, cells were cultured in 96-well microplates (10000 cells/well). The cells were incubated until day 6, 13 or 20 in vitro (DIV). Thereafter, the medium was removed and fresh medium containing different supplements (EPO, Glu) was added. The samples were assayed as described below.

### Incubation with EPO and 1mM Glu under normoxic and hypoxic conditions

To investigate the influence of EPO on cell survival, Glu uptake and GS activity, APC were treated with one of two different concentrations (1 U/ml or 5 U/ml) of human recombinant EPO (Neorecormon, Hoffmann-La Roche, Grenzach-Wyhlen, Germany) and with 1 mM Glu (Merck, Darmstadt, Germany) under normoxic (NC, 10% CO_2_) and hypoxic (HC, 1% O_2_, 10% CO_2_) conditions for 24h.

#### Glutamate uptake

The decrease of the concentration of Glu added to the cell culture media was measured by a direct optical test based on the reaction catalyzed by L-glutamic dehydrogenase (GDH). Cell culture supernatant was replaced at DIV7, 14 or 21 by fresh medium containing 1mM glutamate and 1 U/ml or 5 U/ml EPO, respectively. Subsequently, the cells were incubated for 24h under NC and HC, respectively. Thereafter, 150 µl of supernatant from each well was transferred to a fresh 96-well plate, where it was incubated with the reaction mixture containing 100mM Tris with 100mM hydrazine pH 9.0, 100 mM NAD, 1.7 µl GDH (1500 U/ml, La Roche, Mannheim, Germany) and 3.3 µl H_2_O for 1 h at 37°C. Hydrazine was present in the reaction mixture to pull the equilibrium away from reductive amination. The amount of NADH produced was equivalent to the amount of Glu initially present and detected at 340nm in an ELISA plate reader (Tecan, Grailsheim, Germany). Quantification was performed by six external standard concentrations of Glu in the range between 0-1 mM.

#### Glutamine synthetase activity assay

GS activity was measured by the colorimetric method described by Iqbal and Ottaway [19]. In the presence of ADP, Mn^2+^ and arsenate, GS catalyzes the non-physiological transfer of glutamine to hydroxylamine, yielding gamma glutamylhydroxamate, which forms a purplish-brown complex with Fe(III). One unit of the enzyme was defined as the amount required to catalyze the synthesis of 1 µmol of γ-glutamylhydroxamate per min.

Protein was determined by the method of Lowry, using bovine albumin as a standard. The specific activity of GS is expressed as µmol/min/mg protein.

After incubation of the APC for 24 h under NC and HC with 1mM Glu and with EPO (1 U/ml or 5 U/ml), the cells were washed with ice-cold phosphate-buffered saline (PBS, Biochrom) and lysed with 75µL buffer containing 50 mM imidazole/HCl buffer pH 7.2 (Sigma-Aldrich, Steinheim, Germany). The cell lysis was performed by a freezing-thawing cycle. The reaction catalyzed by GS was initiated by adding 50 µL of the reaction mixture containing 50 mM imidazole/HCl-buffer pH 7.2, (Merck, Darmstadt, Germany), 2mM MnCl_2_, 25mM sodium arsenate, 0.16mM ADP, 50 mM L-glutamine and 25mM NH_4_Cl (Sigma-Aldrich) to the cell lysates. After incubation for 2h at 37°C, the reaction was stopped by 200µL stop solution consisting of 0.37 M FeCl_3_, 0.67 M HCl, 0.2 M trichloroacetic acid (Sigma-Aldrich). Subsequently 300µL of the reaction mixture was centrifuged 5 min at 15000g, 250µl of supernatants were transferred to 96-well plate and measured at 540nm in a microplate reader. The absorbances obtained were converted into concentrations by comparison to an external standard curve made from 10 different concentrations between 0 to 5mM L-glutamic acid gamma monohydroxamate (Sigma-Aldrich).

### Preparation of brain tissue and cell lysates

The brains were processed in ice-cold lysis buffer (300 mmol/L NaCl, 50 mmol/L Tris, 2 mmol/L MgCl2, 0,5% NP40, containing a "Complete Protease Inhibitor Tablet" from Roche Diagnostics at a ratio of 1:4 (tissue weight/buffer volume). The tissue was disrupted using a Micro Dismembrator II (B. Braun, Melsungen, Germany) at maximal amplitude for 30 s in a teflon container which had been precooled with liquid nitrogen. Thereafter, homogenates were clarified by centrifugation at 14000 g and 4°C for 20 minutes. The protein quantification was performed using the BIO-Rad DC Protein Assay (Bio-Rad, Munich, Germany).

APC (n=5) were cultivated in culture dishes (BD, Heidelberg, Germany) under normoxic conditions until day 6, 13 or 20. After treatment with 1 mM Glu under NC and HC for 24h, cells were washed twice with PBS and harvested by scraping into 200 µl PBS followed by centrifugation for 4 min at 14000 g at 4°C. The supernatant was removed by aspiration and the pellet was lysed in 100µl of lysis buffer which contained 50mM Hepes, 150mM sodium chloride, 1.5mM magnesium chloride, 100mM sodium fluoride, 10mM tetra-sodium diphosphate decahydrate, 200µM sodium orthovanadate (Merck), 10% Glycerol, 1% Triton X-100,1mM ethylene glycol-bis (2-aminoethylether)-tetra-acetic acid (EGTA, Sigma, Taufkirchen, Germany) and complete Mini protease inhibitor cocktail (La Roche). The protein concentration in the samples was determined with the Bio-Rad DcProtein Assay.

### Western Blot of GS, EPO and EPOR

The brain tissue or cell lysates were diluted 1:2 with Laemmli buffer and boiled for 10 min at 95°C. Samples containing 50µg of protein were applied to a 12.5% SDS-polyacrylamide gel, electrophoresed and transferred to a PVDF membrane (Millipore, Eschborn, Germany). Membranes were blocked in 0.66% (v/w) I-Block in PBS (Tropix, Applied Biosystems, Weiterstadt, Germany) for 1.5 h and then incubated at 4°C overnight with antibodies against rabbit polyclonal GS (diluted 1:15000, Sigma-Aldrich, Saint Louis, Missouri, USA), mouse monoclonal EPO (diluted 1:500, AbD Serotec, Oxford, UK), rabbit polyclonal EPOR (diluted 1:500, Santa Cruz, CA, USA) and loading control mouse monoclonal GAPDH (1:1000, Millipore, Eschborn, Germany). For visualization of antibody binding, membranes were incubated for 2 h at room temperature with alkaline phosphatase-conjugated goat anti-rabbit or anti-mouse antibody (Tropix), diluted 1:10.000 in I- Block, and thereafter exposed to CDP-Star (Tropix) as chemoluminescence substrate for 1 h in the dark room. Signal intensities were recorded using a CCD camera system.

### Cytotoxicity assay

The cytotoxicity induced by Glu and by hypoxia was determined by measurement of lactate dehydrogenase (LDH) activity in the cell culture supernatants (n=6) of 96-well microplates. The experiments were performed following the protocol indicated in CytoTox 96® Non-Radioactive Cytotoxicity Assay (Promega, Mannheim, Germany). At day 6 or 13 or 20 the medium was renewed and the cells were incubated with 1 mM Glu and 1 U/ml or 5 U/ml EPO for 24h under NC and HC as described above.

To determine LDH 50 µl of the supernatant of each well were transferred to a separate assay plate, mixed with 50 µl Substrate Mix and incubated for 30 minutes at room temperature (RT). The enzymatic conversion of tetrazolium salt into red formazan product was stopped by 50 µl Stop Solution and absorbance was recorded at 490 nm using a 96-well plate reader. Quantification was done by external standardization with LDH concentrations in the range between 0-800 U/ml in DMEM. The absorbance value of a culture medium control was used to normalize the values obtained from the samples.

### Immunofluorescence assay

APC were grown for 6 or 20 days under normoxic conditions. Thereafter, the cells were incubated for 24h with 1mM Glu and/or 5 U/ml EPO under NC and HC. After 24h of incubation, the cells were fixed in -20°C-cold methanol (Merck, Darmstadt, Germany), washed with PBS, and incubated 2h with primary antibodies against Glial Fibrillary Acidic Protein (GFAP, diluted 1:10 with PBS, mouse monoclonal, Progen, Heidelberg, Germany) and Excitatory Amino Acid Transporter 1 (EAAT1, GLAST, diluted 1:100, rabbit polyclonal, Biozol, Eching, Germany] or EPOR (diluted 1:100, Santa Cruz, CA, USA) at RT. For quantification of proliferating cells, methanol-fixed coverslips were washed with PBS and incubated with rabbit polyclonal anti- GFAP (1:500, DacoCytomation, Glostrup, Denmark) and mouse monoclonal anti-proliferating cell nuclear antigen (PCNA) diluted 1:25 (Chemicon, Europe) for 2h at RT. After washing twice with PBS, the cells were incubated in the dark with fluorescein isothiocyanate (FITC)-conjugated goat anti-rabbit IgG (1:100, Dianova, Jackson Immunoresearch, West Grove, USA) and Cy3-conjugated anti-mouse IgG (1:800, Dianova, Jackson Immunoresearch, West Grove, USA).

Thereafter, the cells were washed with PBS containing 0.05% Triton X-100 (Sigma, Steinheim, Germany) and coated with Vectashield mounting medium containing 4´,6 diamidino-2-phenylindole (DAPI, Linaris, Wertheim-Bettingen, Germany) and evaluated by fluorescence microscopy.

The immuncytochemical studies with EPOR-siRNA-treated cells were performed on 13 day-old APC. After 24h incubation with 1mM or 5mM Glu under NC and HC the cells were fixed and incubated 2h with primary antibody rabbit polyclonal anti- EPOR (1:100, Santa Cruz) and mouse monoclonal anti-tubulin β III clone TUJ (1:200, R&D Systems, Wiesbaden-Nordenstadt, Germany) or anti-mouse monoclonal GFAP (dilution 1:10). Thereafter APC were washed twice with PBS and incubated in dark with secondary antibodies FITC (dilution 1:100) and CY3 (dilution 1:800). After washing twice with PBS containing 0.05% Triton X-100 samples were coated with DAPI. The counts of GLAST/ GFAP – and PCNA/GFAP-positive cells were performed from three different experiments (n=5 in each).

For the detection of EPOR in the brain of APP/PS1 mice and their age matched WT-controls, sagittal brain sections (10 µm thick) were fixed in methanol, washed in PBS and stained with rabbit polyclonal EPOR antibody diluted 1:100 (Santa Cruz, CA, USA) at 4°C overnight. Thereafter the sections were washed twice with PBS and incubated in the dark with FITC-conjugated goat anti-rabbit IgG as secondary antibody (1:100, Dianova, Jackson Immunoresearch). The sections were washed twice with PBS+Triton X-100 and coated with Vectashield mounting medium containing DAPI.

All immunofluorescence stainings were evaluated by fluorescence microscopy, using an Olympus BX51 Microscope (Olympus Optical Co. Europe, Hamburg, Germany). Images were acquired by the digital camera F-View II and processed by the software Analysis DOKU® (Soft Imaging System GmbH, Leinfelden-Echterdingen, Germany).

### Reverse transcriptase-polymerase chain reaction (RT-PCR)

APC were plated on 60 x 15 mm culture dishes (n=5) and incubated 6 or 20 days under NC. Thereafter the culture medium was replaced by the fresh medium containing 5U/ml EPO and incubated for 24h under NC and HC. At day 7 or 21, the RNA was isolated using the RNeasy Mini Kit (Qiagen, Hilden, Germany) and quantified by its absorption at 260 nm. RNA quality was assessed by recording the absorbance ratio at 260 and 280 nm [20]. Total RNA (250 ng) was reverse-transcribed in 20 µl reaction mix with AMV reverse transcriptase (PEQ-Lab, Erlangen, Germany), random primers and oligo(dT)15 primer (Promega, Mannheim, Germany). Afterwards, 1µl of this reaction was subjected to qPCR. Reverse transcription was primed by the following primers: EPO Sense: 5´-GGC TGT TGC CAG TGG TAT TT-3´; EPO anti-Sense: 5´-CAC GAA GCC ATG AAG ACA GA-3´.

Amplifications of DNA started with 600 seconds at 95°C and followed with 45 cycles of 95°C for 10 seconds, 68°C for 10 seconds and 72°C for 16 seconds. Real-time PCR assays were performed using the LightCycler® FastStart DNA Master SYBR Green I system in the Light Cycler® 2.0 instrument (Roche Diagnostics, Mannheim, Germany).

### EPO-ELISA assay

APC (6x10^6^ cells/ dish) were plated on 100 x 20 mm culture dishes and grown under NC until day 6 or 20. Thereafter the medium was removed, fresh medium (10 ml/dish) was added and the cells were incubated for 24h or 48h under NC and HC. The supernatant (10 ml/ dish) was collected and EPO concentrations (mU/ml of Medium) were assayed by the EPO-ELISA Medac^®^ kit (Medac GmbH, Hamburg, Germany), according to the protocol of the manufacturer. The concentration of EPO was measured at 405 nm using an ELISA plate reader.

### EPOR siRNA transfection

For siRNA-mediated inhibition of EPOR, a “reverse transfection” protocol” was used according to the manufacturers manual (Qiagen). A master mix for each reaction containing 12.5 ng siRNA EPOR (target sequence AGC CTG TAG TTC CTA AAC CTA), 0.75 µl HiPerFect Transfection Reagent (TR, Qiagen) and DMEM without supplement (total Volume 25 µl for each condition) was prepared and complex formation was allowed to proceed for 10 minutes at room temperature. For determination of cell viability and apoptosis, 25 µl EPOR siRNA-complex was spotted onto each well of a flat-bottom 96-well microplate (white-walled, clear bottom, Greiner, Frickenhausen, Germany), 10000 cells were added to each well to give a final volume of 200 µl and a concentration of 5 nM EPOR siRNA. For determination of glutamate and GS activity, 20000 cells were added to the EPOR siRNA-complex. For immunohistochemical studies and Western blotting, 1x10^6^ cells were seeded into 60 mm culture dishes one day before transfection. After complex formation with HiPerFect, siRNA was added into the plates to give a final volume of 4 ml and a final concentration of 5 nM EPOR siRNA. Negative controls were obtained from untreated cells and those exposed to TR only. Transfection was performed for 48 h under NC and HC. All conditions were run sixfold.

To monitor the gene-silencing effect, the EPOR siRNA-reagent complex was removed and replaced by 150 µl DMEM alone (control) or supplemented with 1mM or 5mM Glu. The effects of Glu on APC were evaluated under NC and HC after 24h incubation.

### Determination of viability and apoptosis

For determination of cell viability and apoptosis, two assays were multiplexed in the same culture well (CellTiter-Blue^®^ and Caspase-Glo^®^ 3/7 Assay, Promega, Mannheim, Germany). For determination of cell viability, 10 µl of the CellTiter-Blue reagent were added directly to each well containing the cells. Incubation was continued for 2 hours to allow viable cells to convert resazurin to the fluorescent resorufin product. The fluorescent signal was monitored at 550 nm excitation and 590 nm emissions using a microplate reader with fluorescence detection (Genios, Tecan, Crailsheim, Germany). The fluorescence value of a culture medium control was used to normalize the values obtained from the samples.

To evaluate apoptosis, caspase-3 and -7 activities were measured. For this procedure, 50 µl of Caspase-Glo 3/7 reagent were added to each well as soon as quantification of cell viability was finished. The assay-plates were incubated for 1 hour at RT and the luminescent signal was measured using a luminescent plate reader (Genios, Tecan, Crailsheim, Germany). The luminescent signal is proportional to the amount of caspase activity, fluorescence of the CellTiter-Blue reagent does not interfere with the assay. The results were expressed as the ratio of apoptosis to viability of the untreated control.

### Statistical analyses

The data obtained from 3 independent experiments for each experimental set (n = 5-6 in each) are presented as mean ± SEM; p <0.05 was considered as significant (*p<0.05, **p<0.01, ***p<0.001). To compare the treated samples with controls and among each other, either Student’s t-test or one-way ANOVA with Bonferroni’s Multiple Comparison Test were used.

## Results

### Expression of GS in astrocytes under hypoxia and exposure to Glu

**Figure 1:**
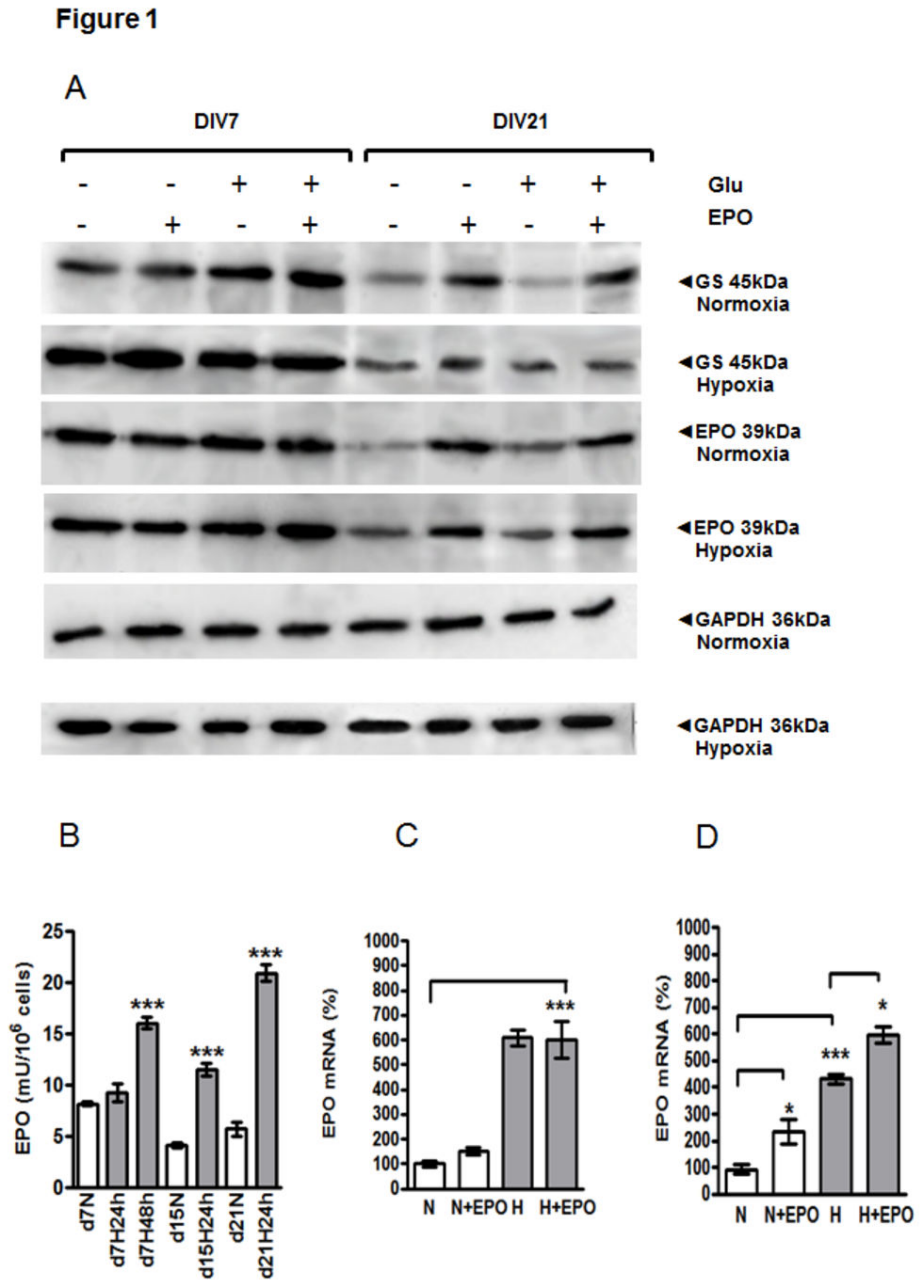
Cell age-dependent effects of glutamate, hypoxia on the expression of EPO, glutamine synthetase (GS), EPO release and EPO mRNA expression in rat APC. (**A**) Western blot analysis of GS and EPO in cell homogenates prepared from 7- (DIV7) and 21-day-old (DIV21) rat APC under normal and hypoxic conditions with or without exposure to Glu and EPO. (**B**) The cell-age-dependent release of EPO into the culture medium under normoxia (N, white bars) and 24h or 48h hypoxia (H, grey bars) in 7-,15- and 21-day old APC. (**C**) The effect of EPO treatment (5U/ml) on the expression of EPO mRNA in early (7-day-old) APC under normoxia (N, white bars) and hypoxia (H, grey bars). (**D**) The effect of EPO on the expression of EPO mRNA in prolonged (21-day-old) APC under normoxic (N) and hypoxic (H) culture conditions. Data are normalized to normoxic control (N) *, p<0.05, **, p<0.01, ***, p<0.001.

Western blot analysis of GS in homogenates of 7-day-old normoxic APC showed an increase of GS in samples from cultures exposed to Glu, either alone or in combination with EPO (Figure 1A, cf. Glu-treated APC at DIV7 under NC with those without Glu). Hypoxia additionally increased the Glu-induced synthesis of GS in 7-day-old APC (Figure 1A, cf. Glu-treated APC at day 7 under normoxia with those upon hypoxia). In aged cultures (DIV21) GS was strongly decreased when compared to young APC (Figure 1A, cf. GS upon normoxic or hypoxic samples at DIV21 with those from DIV7). Hypoxia increased the GS expression in aged APC compared to NC. Both under NC and HC, the GS-inducing effect of Glu was absent from aged astroglial cells. Under NC, Glu even suppressed GS expression in aged astrocytes. This Glu-caused inhibition of GS in aged normoxic cultures was ameliorated by EPO (Figure 1A, cf. GS expression upon normoxia in DIV21 +Glu/-EPO with DIV 21 +Glu/+EPO).

A markedly lower expression of EPO was detected in DIV21 normoxic and hypoxic cultures in comparison to respective normoxic and hypoxic APC from DIV7 (Fig.1A cf. EPO (normoxia) and (hypoxia) of DIV7 with those from DIV21). Hypoxia (24h) enhanced EPO expression in DIV21 APC. Upon Glu exposure, the expression of EPO remained unchanged in both young and aged APC (Figure 1, DIV21 and DIV7 Glu-/EPO- vs. Glu+/EPO-). Interestingly, only on DIV21 the exogenous EPO application was capable of increasing intracellular EPO-content (Figure 1A cf. DIV21-Glu/-EPO with –Glu/+EPO) confirming our data on EPO mRNA detection (Figure 1E)

#### EPO release from APC

The release of EPO was measured in the supernatant by an ELISA at DIV7, 15 and 21 under NC as well as under HC. The comparison between the normoxic control and hypoxia-treated APC at DIV7 revealed no difference when the cells were incubated only 24h under hypoxia (Figure 1B d7N vs. d7H 24h). However, 48h of hypoxia significantly increased the release of EPO from APC (Figure 1B. d7N vs. d7H 48h). At DIV15 the concentration of EPO was decreased under NC in comparison with the normoxic control of DIV7 APC (Figure 1B. d7N vs. d15N 24h). In contrast to DIV7, at DIV15 a significant increase of EPO release was seen already after 24h of hypoxia compared with the normoxic control (Figure 1B. d15N vs. d15H 24h). Similar to the normoxic control of DIV15, at DIV21, a decreased EPO release was measured upon normoxia when compared with the corresponding control from DIV7 (Figure 1B. d7N vs. d21N). The most prominent hypoxia-induced increase in EPO release was detected at DIV21 in comparison with the respective normoxic control (Figure 1B, cf. d21N vs. d21H 24h).

#### Hypoxia- and cell age-dependent expression of EPO mRNA

At day 7 no significant differences in EPO mRNA between control and cells incubated with EPO were observed (Figure 1 C. N vs. N+E, H vs. H+E). Hypoxia induced a nearly 6-fold up-regulation of EPO mRNA when compared to the basal level upon normoxia which was considered as 100% (Figure 1C. N vs. H). In contrast to day 7, at day 21 treatment of APC with EPO led to a significant increase in EPO mRNA levels under both NC and HC, corroborating the results obtained on the protein level by Western blot analysis (Figure 1D. N vs. N+EPO, H vs. H+EPO).

### EPO influence on GLAST

**Figure 2:**
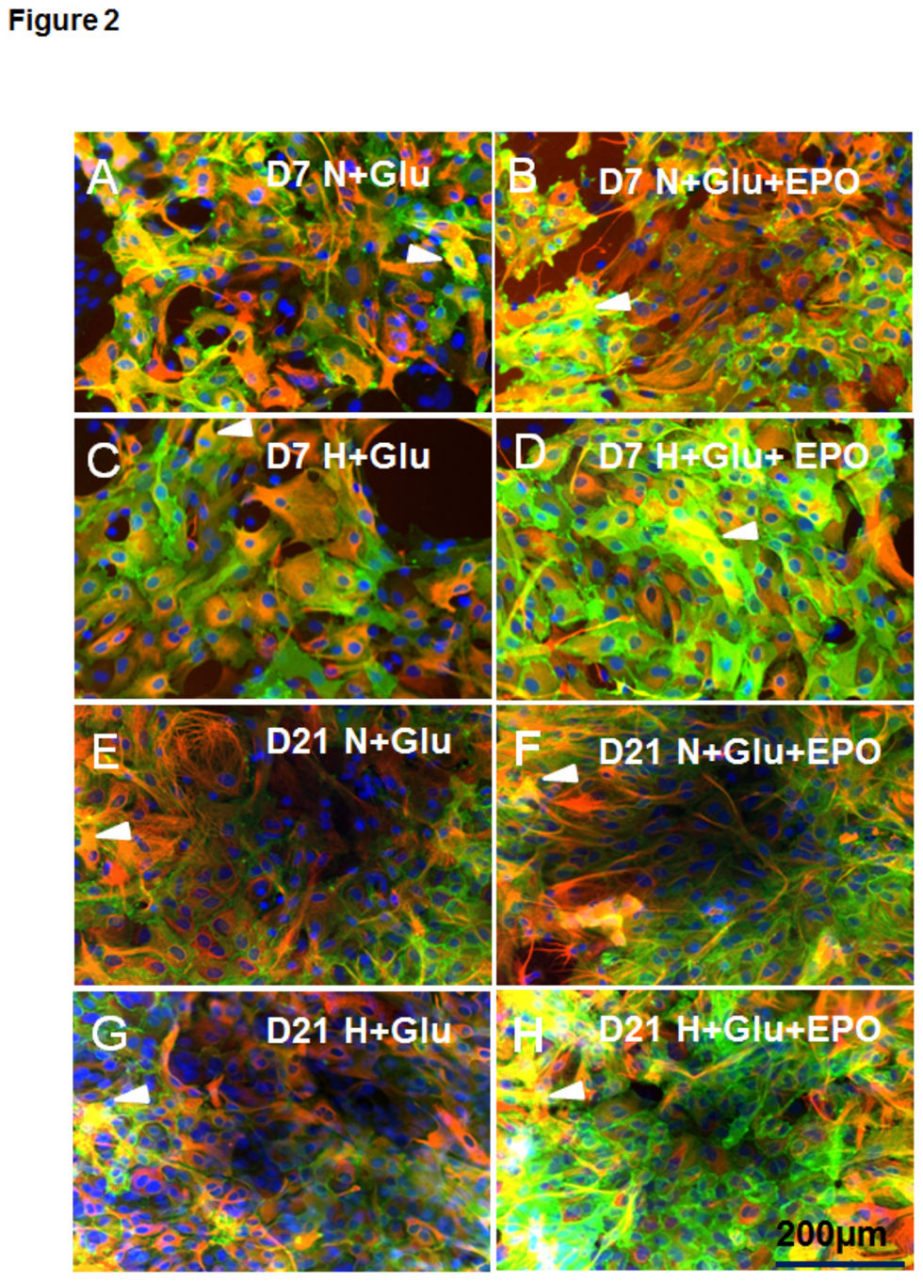
Effects of EPO on the expression of GLAST and GFAP in early and prolonged rat APC treated with Glu under normoxic and hypoxic condition. (**A**) Immunofluorescence analysis of GLAST (green) and GFAP (red) in 7--day old (DIV) normoxic rat astroglial primary cultures upon exposure of Glu; (**B**) Increase in GLAST+GFAP+ cells (yellow cells, arrowhead)after application of 5U/ml EPO upon normoxia (**C**) GLAST/GFAP expression under hypoxia and Glu in DIV 7 APC, yellow cells (arrowhead) are double- positive; (**D**) increased GLAST/GFAP+ cell population (arrowhead) in DIV7 APC after EPO-treatment under hypoxic condition (cf. C vs. D). (**E**) GLAST/GFAP expression in DIV21 APC under NC; (**F**) Exposure of EPO under normoxia led to an increase in GLAST/GFAP+cells (arrowhead, yellow cells). (**G**) APC expressing GLAST/GFAP (arrowhead) upon exposure to hypoxia on DIV21; (**H**) EPO elevated the number of GLAST/GFAP+ in DIV21 upon hypoxia. The cell nuclei were stained with DAPI (blue). Scale bar 200 µm.

**Figure 3:**
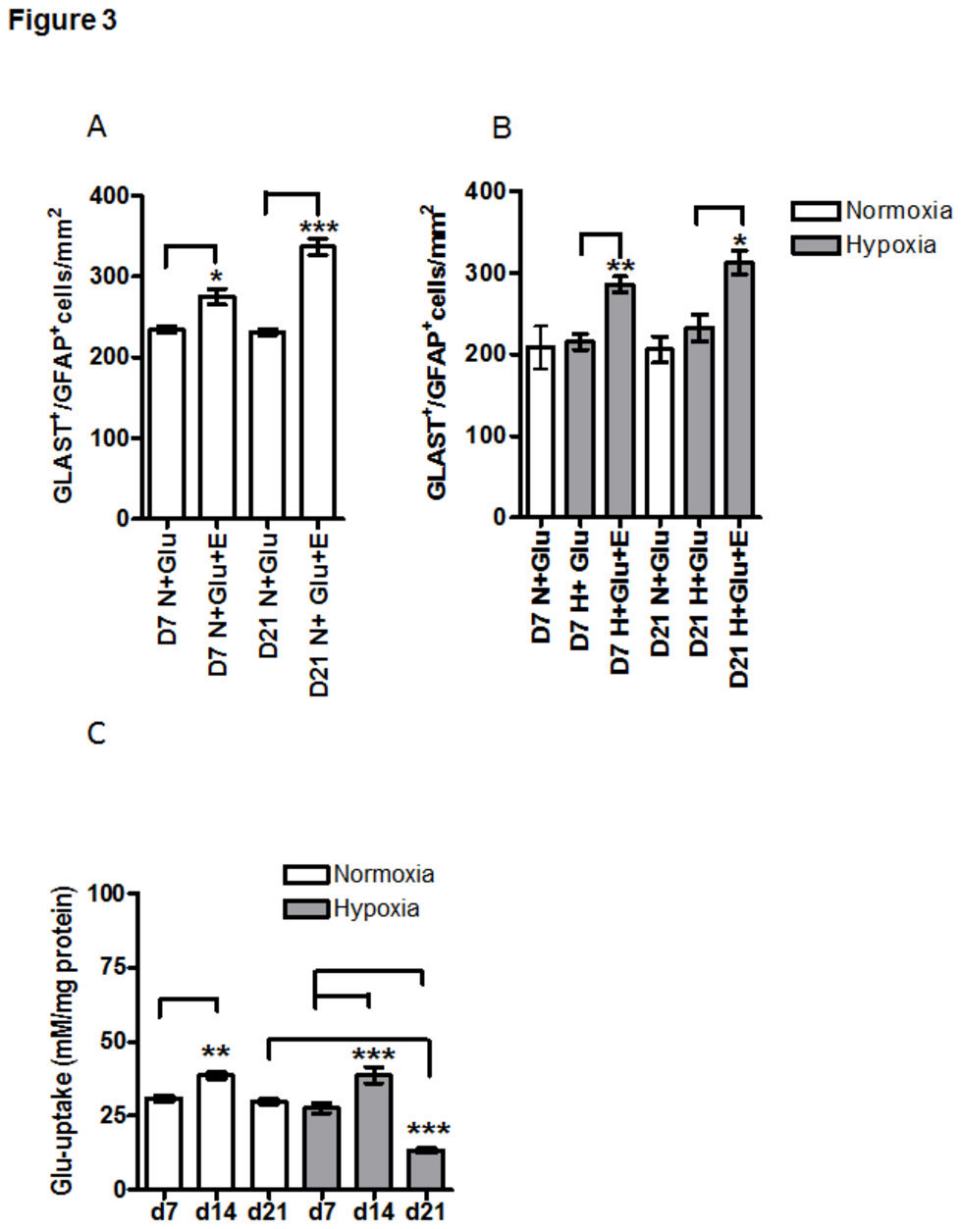
Cell age-dependent Glu-uptake and the effects of EPO on the expression of GLAST by astroglial primary cultures (APC) under normoxic and hypoxic culture conditions. (**A**) Quantification of GLAST-positive astrocytes (GLAST+/GFAP+cells) under normoxia shows a significant (**p<0.01; ***p<0.001) increase in GLAST+/GFAP+cells upon EPO-treatment in both young and aged APC (D7 and D21); (**B**) hypoxia enhanced the effect of EPO on GLAST/GFAP+ cells. In both young and aged cells the number of GLAST/GFAP cells was increased (**p<0.01 at d7 and ***p<0.001 at d21). (**C**) The uptake of 1 mM glutamate by APC (shown in absolute values) was increased on day 14 in comparison with day 7 under normoxia (white bars, **p<0.01) and hypoxia (grey bars, ***p<0.001) and strongly decreased in 21-day old cultures (d21) exposed to hypoxia (grey bars) when compared to those from day 7 in hypoxia (grey bars d21vs. d7, p***<0.001).

Immunostaining of APC exposed to 1mM Glu at DIV 7 showed that the population of GLAST ^+^ /GFAP^+^ cells (yellow cells in Figure 2) was increased after administration of EPO under both normoxia (arrowheads in Figure 2, (A) D7 N+Glu vs. (B) D7 N+Glu+EPO) and hypoxia (arrowheads in Figure 2, (C) D7 H+Glu vs. (D) D7 H+Glu+EPO). At day 21, the application of EPO led also to increase of GLAST ^+^ /GFAP^+^ cells under NC (arrowhead in Figure 2, (E) D21 N+Glu vs. (F) D21 N+Glu+EPO) and under hypoxia (arrowhead in Figure 2, (G) D21 H+Glu vs. (H) D21 H+Glu+EPO). These findings were confirmed by the quantification of GLAST ^+^ /GFAP^+^ cells. The application of EPO in the presence of 1mM Glu increased the number of GLAST ^+^ /GFAP^+^ cells at day 7 and day 21 under both normoxic and hypoxic conditions (Figure 3 A cf. D7 N+Glu vs. D7 N+Glu+E; D7H+Glu vs. H+Glu+E; Figure 3 B cf D7 H+G vs. H+Glu+E; D21 H+Glu vs. H+Glu+E). Moreover, EPO increased the population of GLAST ^+^ /GFAP^-^ cells in all APC investigated under normoxic and hypoxic conditions (cf. green cells in Figure 2 B-H with A-G respectively).

### EPO increases glutamate uptake

**Figure 4:**
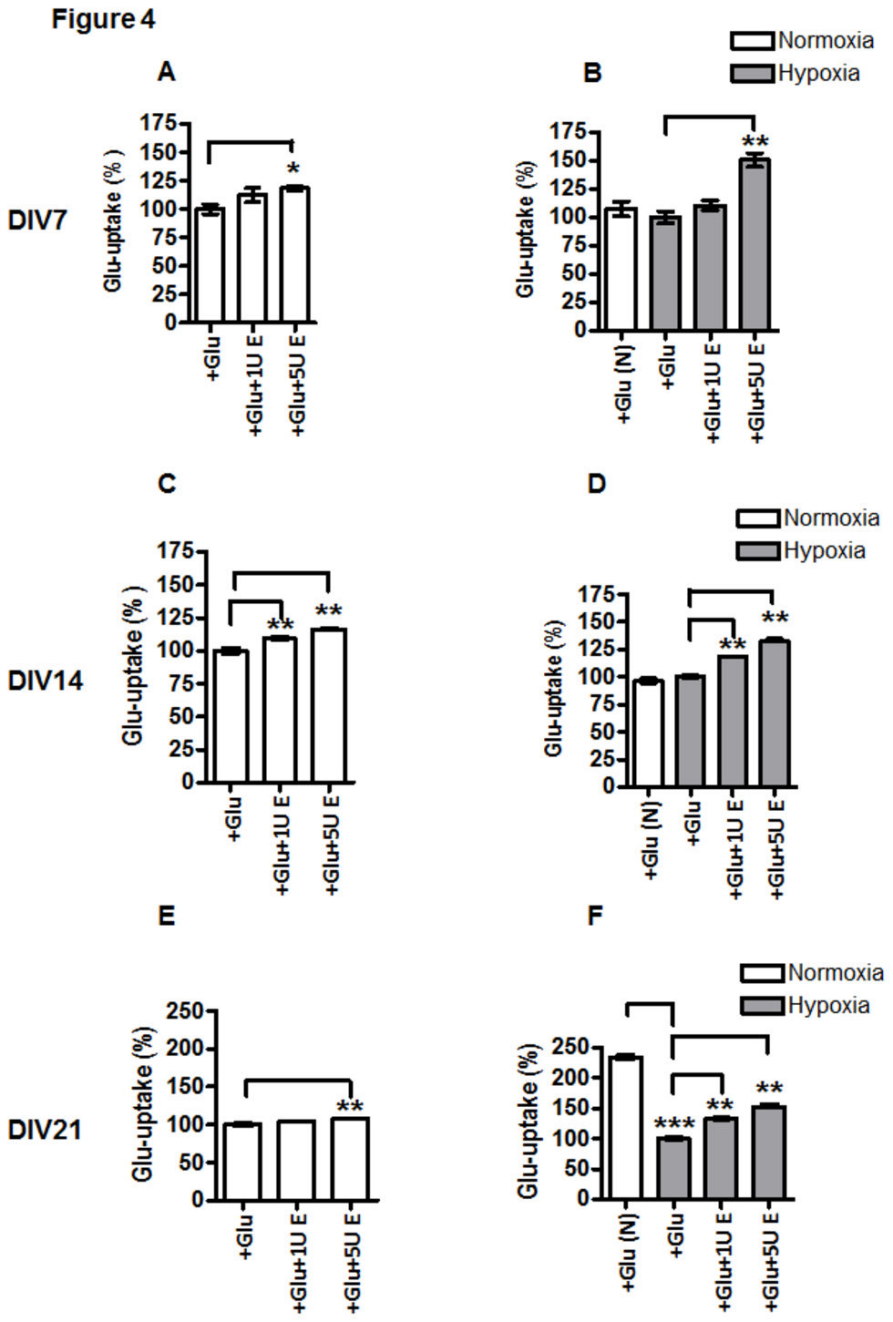
Cell age-dependent and concentration-dependent effects of EPO on Glu uptake by APC under normoxic and hypoxic culture conditions. Glu uptake in APC exposed to 1mMGlu (+Glu in A-F) and 1U/ml EPO (+1UE in A-F) or 5 U/ml EPO (+5U E in A-F). (**A**) normoxic DIV7 APC; (**B**) DIV7 APC under hypoxia; (**C**) DIV14 APC under normoxia; (**D**) DIV14 APC under hypoxia; (**E**) normoxic DIV21 APC ; (**F**) hypoxia-exposed DIV21 APC. The data were normalized to the respective normoxic or hypoxic controls exposed to Glu (+Glu in A-F). Treatment with EPO increased the uptake of glutamate by astroglial cells under normoxic and hypoxic conditions in all cell age groups (D7-D21). This effect of EPO was stronger at concentration of 5U/ml (cf. 1 and 5 U/ml EPO vs. +Glu in A-F) and it increased with time in cultures especially in cultures exposed to hypoxia (cf. B, D and F with A, C and E respectively). Comparison of normoxic (+Glu(N)) vs. hypoxic controls (+Glu) exposed to Glu revealed a decrease in Glu-uptake only in DIV21 APC (F). * p<0.05, ** p<0.01, *** p<0.001.

Treatment of astroglial cells with 1 mM Glu under NC led to a transient increase of Glu uptake at DIV14 (cf Figure 3C d7 with d14).Further, at DIV21, under NC, the uptake of glutamate was equal to that of at DIV7 (cf Figure **3.C** d7 with d21). The Glu uptake by astrocytes at DIV7 and DIV14 was not changed under hypoxic conditions (cf Figure **3C** d7 and d14 normoxia with those upon hypoxia), whereas it was strongly decreased in aged astrocytes, i.e., at DIV21 (cf. Figure **3C** d21 under normoxia [white bar] with d21 under hypoxia [grey bar]). The results shown in Figure **3C** were expressed in mM Glu/mg protein. Further data on EPO effects on Glu uptake were normalized to the respective normoxic control exposed to 1mM Glu (Figure **4**).

To examine the effect of EPO on Glu uptake in astrocytes, we treated APC with EPO (either 1 or 5 U/ml) and 1 mM Glu for 24h under normoxic and hypoxic conditions. The results shown in Figure 4 demonstrated that only a higher concentration of EPO (5 U/ml) was capable of inducing an increase in Glu uptake in all investigated stages of astroglial differentiation, i.e in immature (7 day old), intermediate (14 day old) and aged astrocytes (21-day old APC) upon normoxia and hypoxia (Figure 4 cf. control [+Glu] vs. + Glu+5U E in A-F), while 1U/ml EPO was effective only in intermediate and mature APC, particularly upon hypoxia (cf. +Glu vs. +Glu+1U E in Figure 4 A-F). The data shown in Figure 4 confirm those shown in Figure 3C demonstrating that hypoxia decreased the Glu uptake significantly only in APC on day 21 in comparison with normoxic controls (Figure 4F, white bar +Glu(N) vs. +Glu grey bar) while on DIV7 and 14 Glu-uptake upon normoxic and hypoxic control conditions remained unchanged (cf. white bar +Glu(N) vs. +Glu grey bar in Figure 4B, D and F)

### Astroglial vulnerability to hypoxia and glutamate

**Figure 5:**
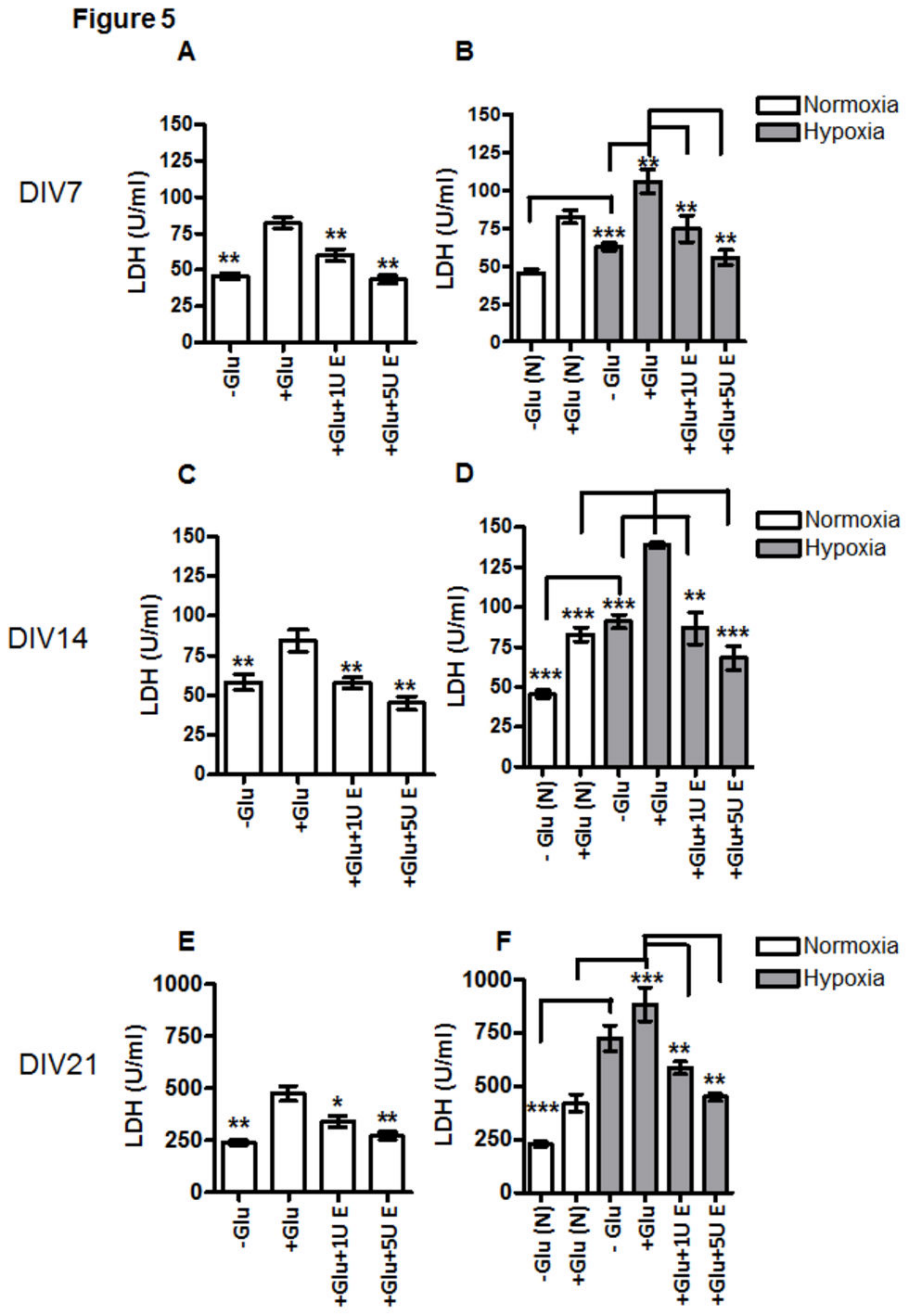
Effect of EPO on glutamate-induced LDH release from APC. (**A**) LDH-release from APC on DIV7 upon normoxia with and without exposure to 1mM Glu (+Glu vs. –Glu) and treatment of 1U/ml (1UE) and 5U/ml (5U E) EPO. (**B**) LDH release from APC on DIV7 upon hypoxia; (**C**) LDH-release from APC on DIV 14 under normoxia; (**D**) LDH-release from APC on DIV14 under hypoxia; (**E**) LDH-release from APC on DIV21 under normoxia; (**F**) LDH-release from APC on DIV21 under hypoxia. Application of 1mM Glu (+Glu in A-F) increased the LDH release from astroglia into the culture supernatant when compared with control without glutamate (-Glu in A-F). *, p<0.05, **, p<0.01, ***, p<0.001.

The vulnerability of APC to Glu is reflected by increased LDH release from the cells both under normoxia and hypoxia conditions at 7, 14 and 21 days compared to the control without Glu (cf. –Glu and +Glu in Figure 5A-F). At day 21 under NC the LDH release from astrocytes, in the absence of Glu, was about 5 fold higher (250U/ml) when compared to that of at day 7 (about 50U/ml, cf. samples without Glu in Figure 5E and A). Treatment of 21-day-old cells with glutamate resulted in nearly a 2 fold increase of LDH in the culture medium (about 500U/ml) when compared to the basal level of LDH (about 250U/ml) in the same age group of untreated astrocytes (cf. samples with Glu in Figure 5E with those without Glu Figure 5E). Hypoxia, by itself, increased the level of LDH in the supernatant of aged cells up to 750 U/ml. Combined exposure of aged cells to hypoxia and Glu resulted in an about 8-fold increase of LDH compared to young 7-day-old astrocytes (cf. +Glu in Figure 5F and B). The highest LDH-release was seen in 21 day old APC exposed to hypoxia (grey bars) with and without Glu when compared with the respective controls upon normoxia (cf. white bars -Glu(N) and +Glu(N) with grey bars -Glu and +Glu in F). EPO abolished the Glu-induced increase in LDH release under normoxia and hypoxia in a concentration-dependent manner: 5U/ml EPO was more effective than 1U/ml (cf. +Glu vs. +Glu+1UE and +Glu+5UE in Figure 5A-F). Administration of 5U/ml of EPO to DIV7 and DIV14 astrocytes under NC and HC resulted in a decrease of LDH activity in cell culture supernatant (cf. samples with +Glu+5U E with samples containing only Glu in A-D). The strongest effect of EPO was observed in aged DIV21 cultures simultaneously exposed to Glu and HC. Treatment with 5U/ml EPO resulted in 2 fold reduction of LDH in supernatants as compared to samples treated with Glu only (cf. +Glu+5U E and samples containing only Glu in Figure 5F).

### EPO increases glutamine synthetase activity

**Figure 6:**
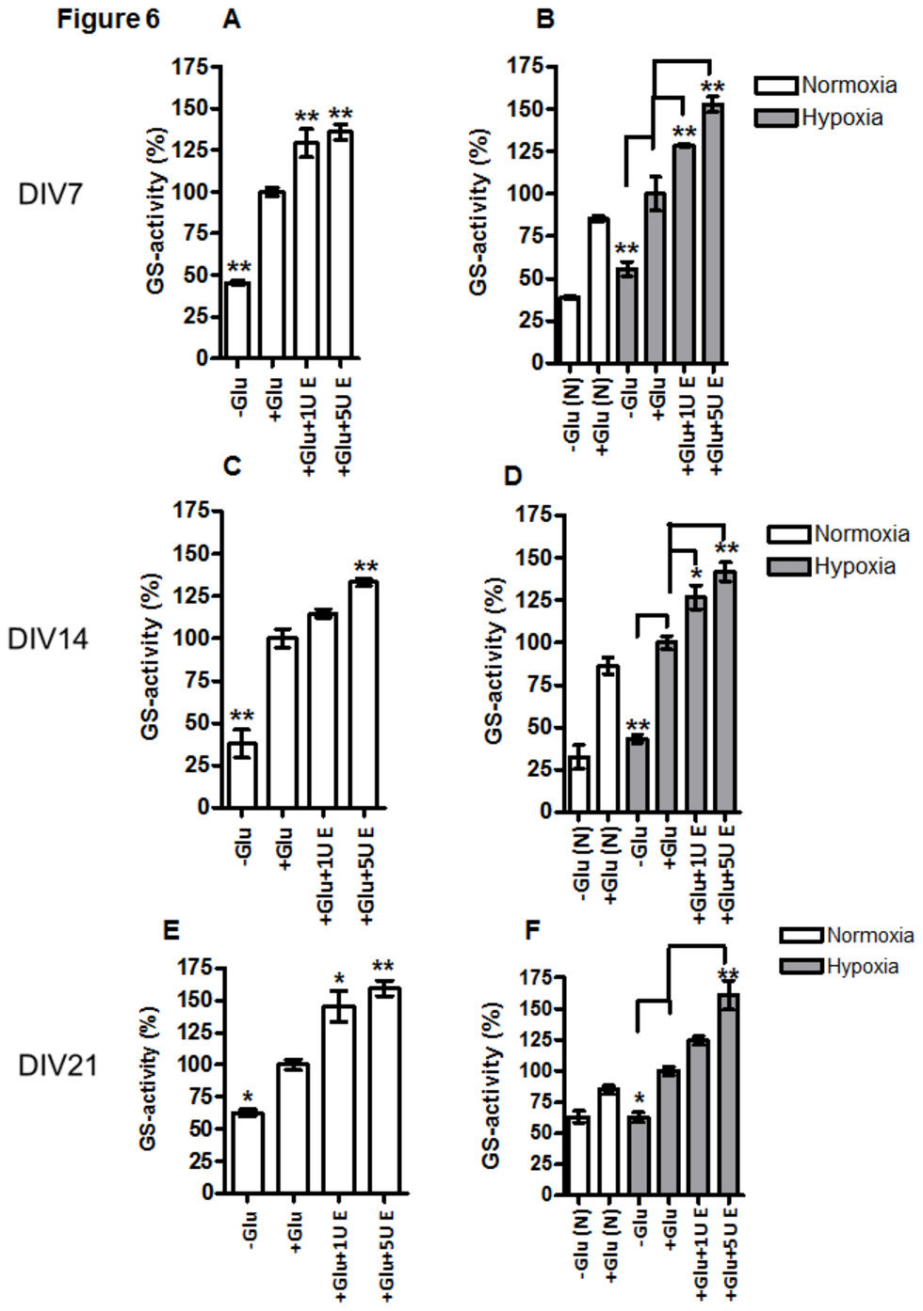
Cell age- and concentration-dependent effects of EPO on glutamine synthetase activity. (**A**) DIV7 APC under normoxia treated with (+1mM Glu) or without (-Glu) and 1 or 5U/ml EPO (1UE or 5 UE respectively); (**B**) DIV7 APC under 24h normoxia /white bars) or hypoxia (grey bars) treated with (+Glu) or without (-Glu) and 1 or 5U/ml EPO (1UE or 5 UE respectively); (**C**) DIV14 APC under normoxia treated with (+Glu) or without (-Glu) and 1 or 5U/ml EPO; (**D**) DIV14 APC upon 24h normoxia /white bars) or hypoxia (grey bars) treated with (+Glu) or without (-Glu) and 1 or 5U/ml EPO (1UE or 5 UE respectively); (**E**) DIV21 APC under normoxia treated with (+Glu) or without (-Glu) and 1 or 5U/ml EPO (1UE or 5 UE respectively); (**F**) DIV21 APC under 24h normoxia /white bars) or hypoxia (grey bars) treated with (+Glu) or without (-Glu) and 1 or 5U/ml EPO (1UE or 5 UE respectively). At all three time points in culture, Glu increased the activity of GS when compared to respective controls culture without Glu (-Glu). Treatment with EPO increased the glutamate-induced activation of GS in concentration-dependent manner when compared to control culture (cf. +Glu vs +Glu+1U E and +Glu+5U E). *, p<0.05, **, p<0.01, ***, p<0.001.

GS-activity was not affected by exposure of APC to hypoxia on day 7, 14 and 21 (cf. white bar –Glu(N) with grey bar –Glu in Figure 6B, D and F). Glutamate increased the GS activity in APC in all investigated stages of astroglial differentiation (DIV 7, 14 and 21), under both NC and HC (cf. –Glu with +Glu in Figure 6 A-F). Administration of EPO (1 or 5 U/ml) caused an additional increase in GS activity in Glu-treated APC (Figure 6A-F, cf. +Glu with +Glu+1U E or with +Glu+5U E) with an exception of 21-day old APC exposed to 1U/ml EPO upon hypoxic condition and 1mM Glu (Figure 5F). These results also show that generally the GS activity in APCs was more effectively increased by 5U/ml than by 1U/ml EPO, particularly in 14 and 21-day old APC upon hypoxia when compared with the control exposed to Glu only (cf. +Glu vs. +Glu+5UE in Figure 5D and F).

### EPOR controls the viability, Glu uptake and GS activity in astroglia

**Figure 7:**
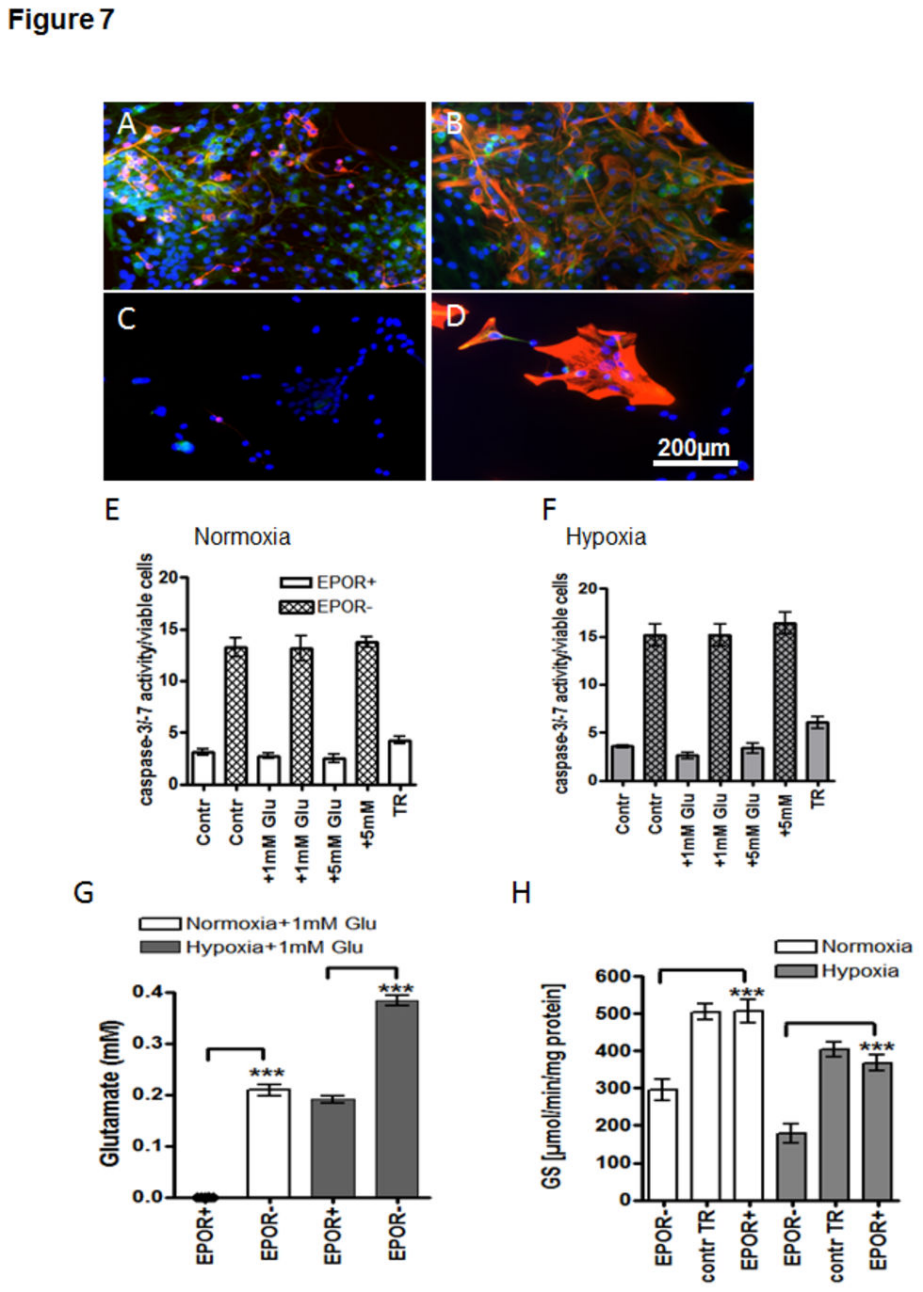
The significance of EPOR for astroglial cell survival and utilization of glutamate by astroglial cells. (**A**) Immunostaining for EPOR (green) and β-tubulin III (red) in DIV14 APC; (**B**) Immunostaining of EPOR (green) and GFAP (red) of DIV14 APC; (**C**) Immunostaining for EPOR (green) and /ß-tubulin III (red) in DIV14 APC transfected with EPOR siRNA; (**D**) Immunostaining of and EPOR (green) and /GFAP (red) of 14-day-old APC transfected with EPOR siRNA. Cell nuclei are stained with DAPI shown in blue. Scale bar 200µm. (**E**) Caspase 3/7 activity in untreated 14-day-old APC (white bars) and those transfected with siRNA for EPOR upon normoxia (patterned white bars) (**F**) Caspase 3/7 activity in hypoxic untreated 14-day-old APC (grey bars) and those transfected with siRNA for EPOR (patterned grey bars) Treatment of original APC (EPOR+) with transfection reagent (TR) did not influence significantly the survival of APC under both normoxic (white bars) and hypoxic (grey bars) conditions. (**G**) Glu uptake in untreated (EPOR+) APC at DIV14 and those transfected with EPOR siRNA (EPOR-) under normoxia (white bars) and hypoxia (grey bars) (**H**) GS-activity in untreated (EPOR+) APC at DIV14 and those transfected with EPOR siRNA (EPOR-) under normoxia (white bars) and hypoxia (grey bars). Treatment of original APC (EPOR+) with transfection reagent (contr TR) did not influence significantly the GS-activity of APC. * p<0.05, ** p<0.01, *** p<0.001.

Silencing of EPOR in 14-day old APC (EPOR^-^APC) resulted in dramatic decrease in total cell numbers (cf. DAPI+ blue cells in A, B vs. C and D in Figure 7) as well as in disappearance of the β-tubulin III- positive, neuronal cell population (cf. red cells in Figure 7A and C), and decreased the number of GFAP+ astroglial cells in APC (cf. red cells in B vs. D in Figure 7). Effective EPOR silencing by siRNA transfection is reflected by the absence of EPOR-positive cells in 14-day old APC (green cells in Figure 7 C and D). A dramatic (4-5-fold) increase in the number of apoptotic cells was observed after EPOR-siRNA transfection of APC (patterned bars in Figure 7 E-F) under both normoxia and hypoxia when compared with the respective untreated or transfection reagent (TR)-treated controls (white and grey bars in Figure 7 E-F). Under both normoxia and hypoxia, different concentrations of Glu (1 or 5 mM) did not additionally increase the number of apoptotic cells in APC (cf. Contr. EPOR- with +1mM Glu EPOR- or +5mM Glu EPOR- in Figure 7 E and F). Quantification of Glu in the cell culture supernatant 24h after administration of 1mM Glu revealed an impaired Glu uptake in cultures transfected with EPOR-siRNA. As shown here upon normoxia, untreated APCs nearly completely utilized 1mM Glu after 24h, while 0.2 mM Glu was still present in supernatants from EPOR(-)- cells (cf. EPOR(+) vs. EPOR(-) white bars in Figure 7G). This may be explained by the existence of alternative EPOR-independent pathways that partially control Glu-uptake in astrocytes. Hypoxia led to impaired utilization of Glu already in untreated control (cf. white EPOR+ bar with grey EPOR+ bar Figure 7G) which was further potentiated by EPOR silencing (cf. grey bars EPOR(+) vs. grey patterned bars EPOR(-) in Figure 7G). However, even after EPOR silencing astroglia retained a residual capacity to metabolize Glu as evidenced by presence of 0.4 mM Glu in supernatants from EPOR (-)- cells treated with 1mM Glu (grey EPOR(-) bar in Figure 7G).

EPOR silencing dramatically decreased the GS-activity (cf. EPOR- vs. EPOR+ in Figure 7 H) under normoxic (white bars in Figure 7H) and hypoxic (grey bars in Figure 7H) conditions.

### EPOR expression in young and aged APC upon normoxia and hypoxia

**Figure 8:**
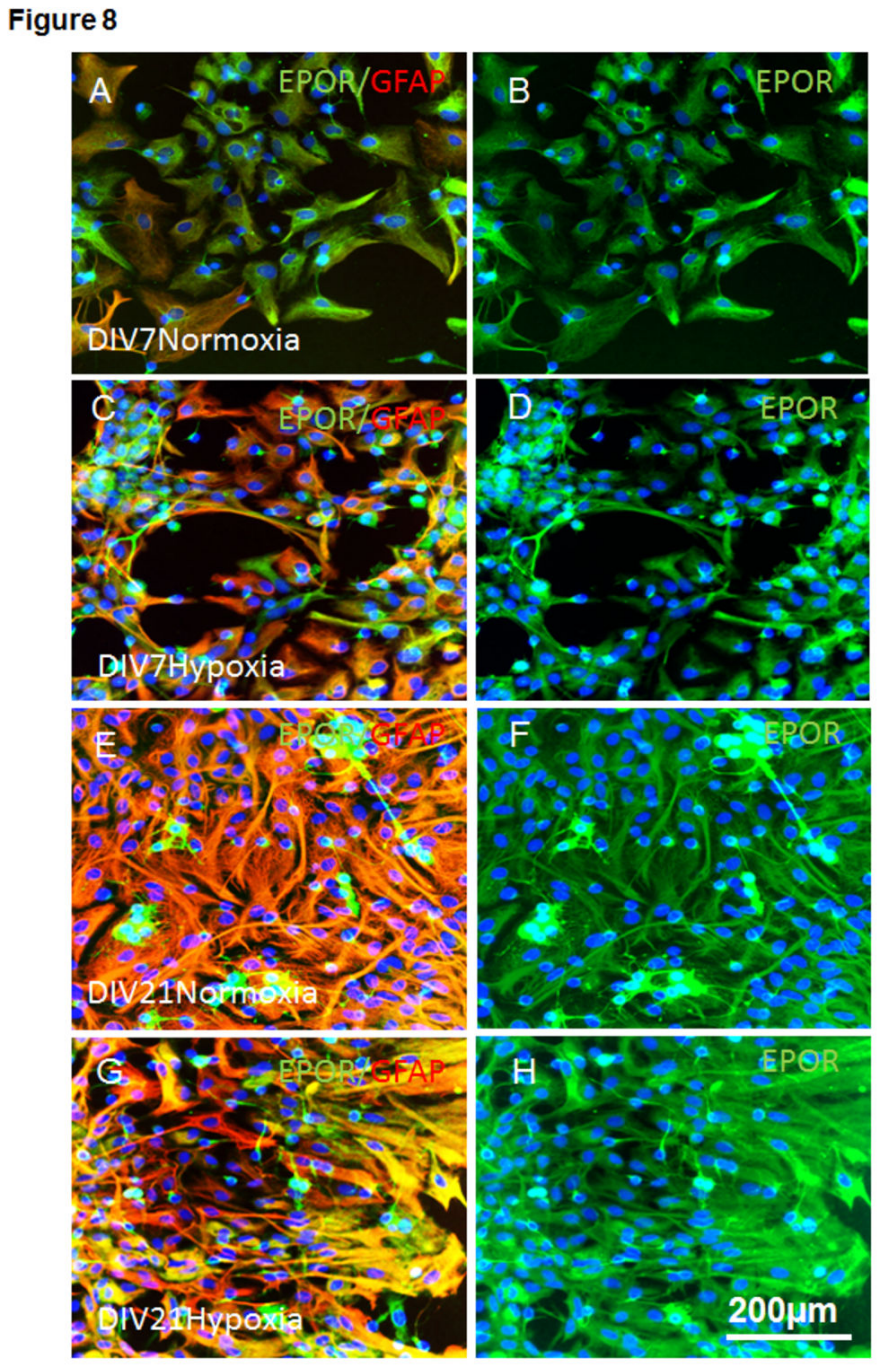
Expression of EPOR/GFAP in young and culture aged APC under normoxia and hypoxia. (**A**) Combined picture of double immunostaining of EPOR (green) and GFAP (red) in 7-day old APC under normoxia; (**B**) Corresponding picture of EPOR in green; (**C**) Double immunostaining of EPOR (green) and GFAP (red) of APC DIV 7 upon hypoxia; (**D**) Corresponding picture to C of EPOR (green) only; (**E**) EPOR (green) and GFAP (red) expression in APC on DIV21 under normoxia; (**F**) EPOR (green) picture corresponding to E; (**G**) EPOR(green) and GFAP(red) expression in APC at DIV21 upon hypoxia; (**H**) corresponding to G picture of EPOR (green) only in DIV21 APC under hypoxia. A-H Nuclear staining with DAPI shown in blue, scale bar 200µm.

A moderate expression of EPOR was detected in young APC (DIV7), while aging in culture led to a marked upregulation of EPOR and GFAP under both normoxic and hypoxic conditions in DIV21 APC (cf. A-D for DIV7 with E-H for DIV21 in Figure 8). EPOR and GFAP expression was further enhanced by exposure of young and aged APC to hypoxia (cf. DIV7 Normoxia (A-B) vs. Hypoxia (C-D) and DIV21 Normoxia (E-F) vs. Hypoxia (G-H) in Figure 8)

### EPO increases proliferation of APC under hypoxia

**Figure 9:**
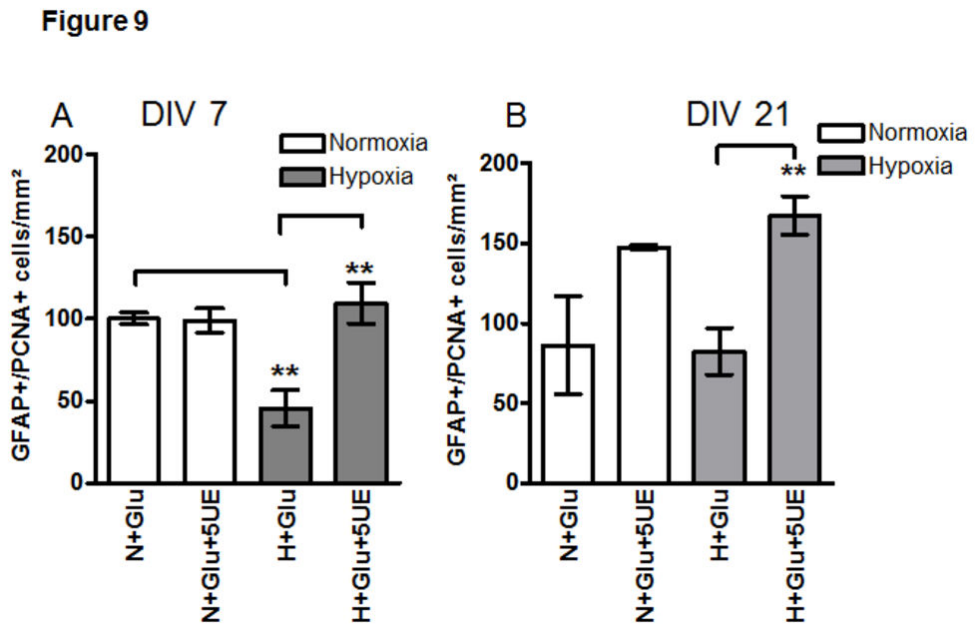
Quantification of GFAP/PCNA-positive cells in APC in young and culture aged APC upon hypoxia/Glu/EPO-exposure. (**A**) GFAP/PCNA+ cell counts in APC at DIV7 exposed to Glu under normoxia (N, white bars) and hypoxia (H, grey bars) showed an increase of proliferating astrocytes upon exposure to 5U/ml EPO (5U E) only under hypoxic conditions. Hypoxia decreased the proliferation of APC (cf- N+Glu vs. H+Gu); (**B**) Quantification of GFAP/PCNA+ cells on DIV21 shows no changes in PCNA+ astrocytes exposed to EPO under normoxia (white bars), while a significant increase (**p<0.01) is detected in EPO-treated APC (H+Glu+5U E) under hypoxia (grey bars), as compared with the hypoxic control (H+Glu). The proliferation of APC in normoxic (N+Glu) and hypoxic control conditions (H+Glu) remained unchanged.

Quantification of GFAP+/PCNA+ cells in young and old APC exposed to Glu showed a decrease in the population of proliferating APC under hypoxia in young APC (Fig.9 AN+Glu vs. H+Glu), while in old cultures the number of GFAP/PCNA+ cells remained unchanged (Figure 9 B, N+Glu vs. H+Glu). Under normoxia EPO slightly, albeit not significantly increased the proliferation of astroglia only in old APC (Fig.9A N+Glu vs. N+Gu+5UE and Fig.9B N+Glu vs. N+Glu+5UE). In contrast, exposure of EPO to APC under hypoxia led to a significant increase in PCNA+ cells in both, young and old APC (cf. Figure 9A and B, H+Glu vs. H+Glu+5UE).

### Age-dependent expression of EPO and EPOR in the brains of APP/PS1 and normal mice

To investigate the age-dependent expression of EPO and EPOR in vivo during normal aging and in Alzheimer’s-like neurodegeneration we tested their expression in the brains of APP/PS1 mice and age-matched wild type controls.

**Figure 10:**
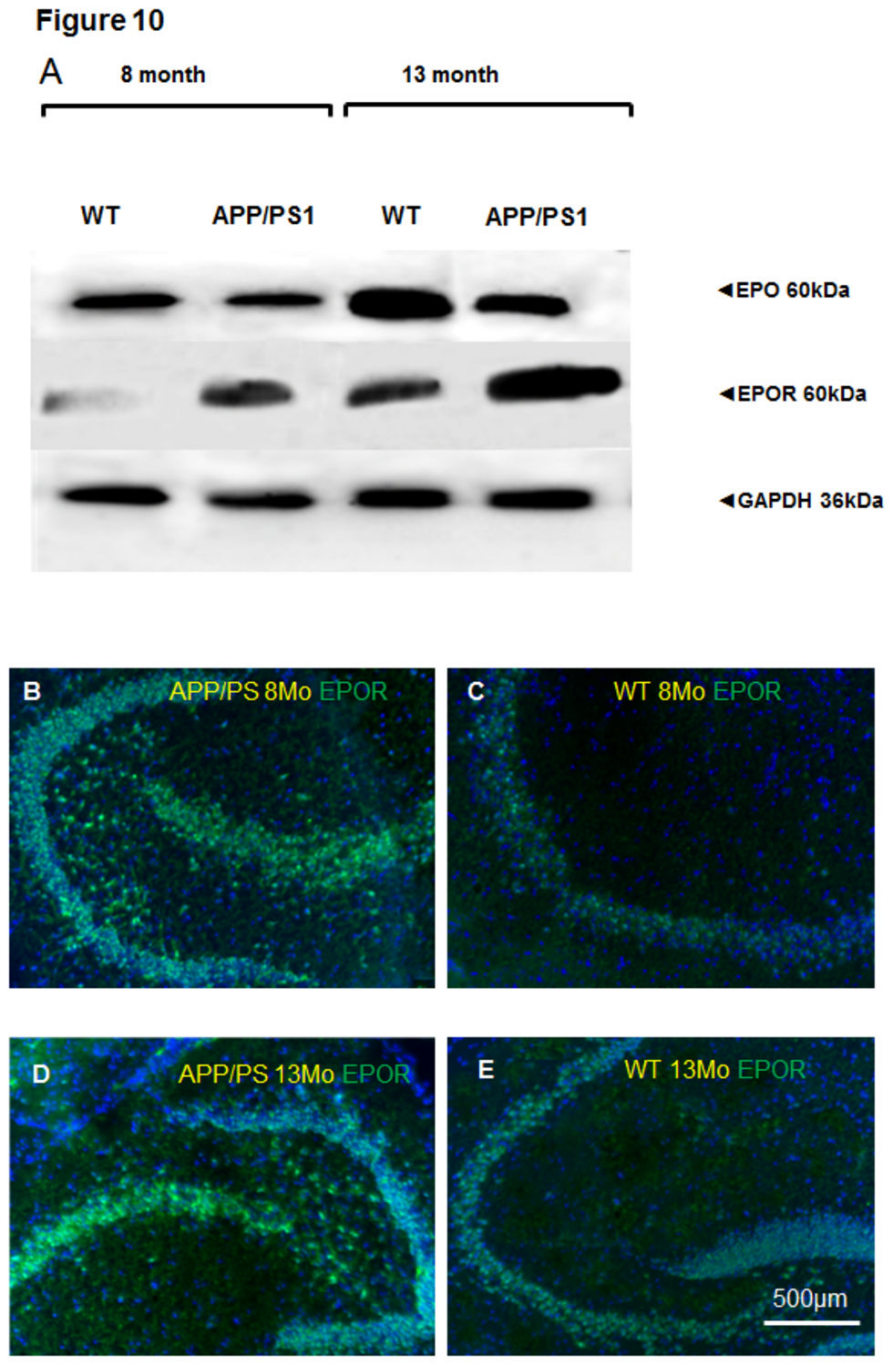
EPO and EPOR expression in 8- and 13 month old APP/PS1 and WT mice. (**A**) Western blot analysis of EPO and EPOR in brain homogenates from 8- and 13-month-old APP/PS1 and WT mice. GAPDH serves as a loading control (**B**) Representative immunostaining of EPOR in the hippocampal area of 8-month old APP/PS1 mouse; (**C**) EPOR expression in 8 month old WT mouse hippocampus; (**D**) EPOR (green) in 13 month old APP/PS1 mouse hippocampus; (**E**) Corresponding WT-control to D showing EPOR (green) in 13 month old WT mouse hippocampus. Scale bar 500µm.

Both, the immunofluorescence analysis of the hippocampus and Western Blots of whole brain homogenates showed a prominent increase in expression of EPOR in APP/PS1 mice when compared with their age-matched WT-controls (cf. EPOR in APP/PS1 vs. WT in Figure 10 A, B vs. C and D vs. E). Aging increased EPOR in APP/PS1 and WT mice reflected by its higher expression in 13 month old mice, when compared with 8 month old animals (cf. APP/PS1 13 month with 8 month and WT 13 month vs. 8 months in Figure 10 A and B vs. D and C vs. E).

It is not surprising that EPO and EPOR expression were directly correlated: the younger the animals the lower the expression of EPO in both APP/PS1 and WT mice.

Additionally, EPO expression was generally lower in APP/PS1 mice as compared to their age-matched WT controls.

## Discussion

In view of the strong neuroprotective effect of EPO in different types of neurodegeneration (for review, see 15), we proposed that the neuroprotective potency of EPO can be partially ascribed to its capacity to control astroglial Glu metabolism and the vulnerability of astroglia to the deleterious environment created by hypoxia and Glu excess. To test this hypothesis, the dynamics of EPO effects on mechanisms responsible for removal and metabolism of exogenous Glu during in vitro aging of astrocytes has been investigated both under hypoxic and normal culture conditions.

An increasing body of evidence shows that the in vitro aging of astrocytes is accompanied by morphologic and biochemical differentiation, resulting in acquisition of features proper for astrocytes in senile and degenerated brain. In our previous [21] and present study b**y** using long-term astroglial cultures as a model of astrocyte aging it has been shown that until day 7 in culture, the most of cells show flat cobblestone-like morphology. At day 14 there was a strong morphologic heterogeneity among astrocytes: some cells showed flat or cobblestone-like morphology whereas others developed an arborisation pattern. The overall morphologic and antigenic differentiation of astrocytes, i.e. development of fine processes strongly labelled with antibodies against GFAP, took place at day 21 in culture and warrants labelling them "aged" cells. Culture-aged astrocytes were more sensitive to hypoxia and Glu. Morphologic differentiation of astroglial cells after 21 days in culture may include changes in antigenic composition. In the present study, the antigenic differentiation of aged astrocytes is confirmed by down-regulation of GS and GLAST expressions examined by WB and double immunolabelling, respectively. Furthermore, in culture-aged astrocytes Cd,Zn-metallothionein I+II (MT), small oxygen-sensing cysteine-rich EPO-inducible proteins [22] presenting a powerful defence against reactive oxygen species (ROS) and free radicals are down-regulated [21,23]. This may explain the high sensitivity of aged astrocytes to hypoxia and effects of EPO in prolonged normal and hypoxic culture conditions shown herein.

Hypoxia suppresses Glu transport in astrocytes and therefore promotes extracellular accumulation of glutamate [24], impairment of mitochondria activity, accompanied by loss of Ca^2+^ homeostasis and generation of ROS [25].

When considering EPO as a measure of safety and protection against variety of neurotoxic insults such as hypoxia, glutamate toxicity [26] and free-radical injury, the level of EPO during deviations from physiological to early pathological situations may be considered as indicator of functionality of astrocytes. As shown here, young astroglial cells show higher levels of EPO expression and release under normoxic conditions when compared to culture-aged cells. Treatment of culture-aged cells with glutamate under NC led to down-regulation of GS evidenced by Western blot, which could be reversed in the presence of EPO. This suggests that under normal oxygen tension, exogenously added EPO can trigger the production of endogenous EPO and GS in astroglial cells.

In accordance with Masuda et al. [12], in our study, RT-PCR analyses indicated that the regulation of EPO production by oxygen operates at the level of its mRNA. This response of astroglial cells is much stronger in young cultures when compared to that of in culture-aged cells. As expected in young astrocytes, the basal expression level of EPO mRNA as well as the striking 6-fold hypoxia-induced up-regulation of EPO mRNA transcription could not be further modulated by administration of EPO, suggesting a sole role for hypoxia in regulation of EPO levels in young cells. Liu et al. [27] showed increased EPO mRNA expression in astrocytes cultured under 5% O_2_, as compared to cells cultured under normoxic conditions. In the same study administration of EPO to superoxide dismutase 2 (SOD2)^-/-^ mouse astrocytes characterized by excessive generation of reactive oxygen species improved the survivability of these cells under normal in vitro conditions. Further confirmation of ROS-related effects of EPO was obtained by administration of EPO to heterozygous (SOD2)^-/+^ mutant mice 24h before exposure to paraquat, a reactive oxygen species generator. EPO increased the survival of the animals. These effects of EPO are probably due not only to activation of Jak-STAT signal transduction pathways [27], but also due to induction of metallothionein [22], a powerful scavenger of ROS.

Our study demonstrates that EPO gene expression depends not only on hypoxia but also on the level of EPO in the environment. The fact that hypoxia-induced up-regulation of EPO mRNA in young cells could not be further influenced by EPO may be explained by the notion that in young cells endogenous EPO level induced by hypoxia is sufficient for achievement of maximal increment of EPO mRNA. Since EPO is among the target genes of hypoxia-inducible factor (HIF), it is conceivable that decreases in EPO mRNA level in aged astroglial cells under hypoxia are associated with cell age-related down-regulation of HIF-1alpha and impairment of HIF-dependent gene expression demonstrated in in vivo studies [28,29] Furthermore, treatment of aged cells with EPO induced a 2.5- and 1.4- fold increase of EPO mRNA under normoxia and hypoxia, respectively. This can be explained by the proliferation-inducing effect of EPO on astroglia, which is further confirmed by quantification of GFAP/PCNA-positive cells. Astroglial proliferation under EPO in aged cells (d21) leads to an increase in the population of young cells. As it is shown in Figure 1 A and B, young astroglia possesses a stronger capacity of expressing and releasing EPO. The increase in the population of young cells in aged 21-day old culture exposed to EPO leads to increased total levels of EPO mRNA and protein. These findings confirm again our previous work showing a rejuvenating effect of EPO on astroglia reflected by increased population of GFAP/nestin-positive young astrocytes and slowed astroglial differentiation [17].

Western blot analysis of GS in homogenates of young and aged astrocytes revealed cell age-related alterations in the glutamate-metabolizing activity of astrocytes. Treatment of young astrocytes with 1mM Glu under NC resulted in a strong up-regulation of GS protein, a reaction that was exacerbated under HC. Addition of EPO slightly increased this protective response of young cells under HC. This reaction of astroglial cells to hypoxia was reminiscent of the one observed in rat pheochromocytoma cells. Also in these cells, GS mRNA and protein were increased upon exposure to hypoxia [30]. The high expresssion of GS in 7-day-old primary astroglial cells under NC and especially under HC implies their capacity for interaction with neurons via the glutamate-glutamine cycle even under pathological conditions. Furthermore it suggests that GS belongs to hypoxia-inducible proteins, similar to EPO and MT. As expected, treatment of young astrocytes with EPO increased the glutamate-induced enzymatic activity of GS in a concentration-dependent manner. It has been previously shown that the magnitude of GS reduction by hypoxia in astrocytes depends on the age of cells in culture [11]. Our data show that GS-activity was not affected by exposure of APC to hypoxia on day 7, 14 and 21, while Glu prominently increased the GS-activity upon normoxia and hypoxia in all culture ages investigated. Tholey et al. [11] reported a decrease in GS activity in APC after 9h hypoxia (without Glu) at day 10 and 14 in culture, while at day 5 the GS activity remained unchanged under hypoxia. However, the hypoxic conditions used in the cited study can be described as anoxic rather than hypoxic (5% CO2/95%N2, no oxygen), which is likely to severely affect the survival and Glu-metabolizing function of astrocytes. Twenty four hours under such condition resulted in total detachment and death of astroglial culture [31]. It can be assumed that Glu-uptake and metabolism of astrocytes depends not only on age but also on severity and duration of oxygen deprivation. This notion is supported by the work of Sher and Hu [32], who see no changes in GS-activity in 12-day old brain primary cultures containing neurons and astroglia upon moderate hypoxia (5% O2) without Glu for 24 h (similar to our results), while under 48h of hypoxia the GS-activity level was even increased.

Under hypoxia, EPO increased the enzymatic activity of GS in aged glutamate-treated astrocytes. This may be due to a reduction by EPO of the factor detrimental to GS activity, oxidative stress. It is known that GS is a particularly vulnerable target for oxidation in the CNS [33], and that EPO can reduce oxidative stress in the brain [34].

In accordance with other reports [30], in our study the enzymatic activity of GS was upregulated during simultaneous exposure of APC to hypoxia and Glu with increasing time in culture. On the other hand, as evidenced by Western blot analysis, the GS protein level in aged astrocytes decreased. Such complicity in GS regulation we came across also in our previous study performed on GFAP-positive keratinocytes of the skin [35], i.e. exposure of primary keratinocyte cultures or cell lines to ammonium ions resulted in enhanced enzymatic activity of GS, but not GS protein expression.

Glutamate uptake by astrocytes is fundamentally important in the regulation of CNS function. Disruption of uptake can lead to excitotoxicity and is implicated in various neurodegenerative processes**.** Consistent with the findings of Dallas et al. [24], **i**n our study the Glu uptake under HC was decreased in aged cells. As shown here the capacity of culture-aged astrocytes for glutamate uptake was significantly decreased under HC, and it was slightly impaired even under NC in comparison with young cultures. The compromised adaptation of Glu-uptake by aged astrocytes may serve as an additional marker of their senescence. Various stress factors were shown to induce the senescent phenotype of astroglial in culture. Inflammation reflected by release of inflammatory interleukins and chemokines including IL-1β and monocyte chemoattractant protein-1 (MCP1) is the one of these factors. Since hypoxia induces the production of IL-1β and MCP1 in astrocytes [36,37] it can be assumed that oxygen deprivation serves as a factor triggering the senescent pehotype of astrocytes. Though EPO at both concentrations (1U/ml and 5U/ml) increased the Glu uptake in intermediate and mature stage of astrocytes, its strongest effect occurred in aged astrocytes and using 5U/ml EPO.

The efficiency of EPO at concentration of 5U/ml seems to be not a random phenomenon. This concentration of the EPO was measured in cerebrospinal fluid (5.148 U/ml) of stroke patients after intravenous administration of 33,000 IE of human recombinant erythropoietin resulting in significant improvement of clinical outcome and neurological recovery [16]. At concentration of 5 U/day EPO was shown to induce the formation of synapses within different brain layers of gerbils and it protected primary cultured hippocampal and cerebral cortical neurons from NMDA receptor-mediated glutamate toxicity [26].

Malfunction of astrocytic Glu transporters will lead to an excessively high extracellular Glu concentration which may result in neurodegeneration caused by the excitotoxic action of glutamate [2]. Hypoxia suppresses the Glu uptake by astrocytes and expression levels of GLAST [24,38]. Here we show that EPO is capable of increasing the population of GLAST+ astrocytes. Moreover, this effect of EPO was again more pronounced in aged astrocytes exposed to Glu (Figure 3 A, B). The mechanism of glutamate transporter suppression by hypoxia has been ascribed to NF-κB activation [24]. However, the dual role of NF-κB under hypoxia raises the question whether its activation is protective or cytotoxic for neural cells as well as to which extent its activation versus inhibition is involved in EPO effects on astroglia. NF-κB activation was reported to decrease mammalian cell apoptosis and to be an essential pathway for EPO effect on neuronal survival and generation of neural stem cells (for review, see 39). The opposite scenario of a GLAST expression-enhancing effect of EPO relies on the study showing its inhibiting effect on NF-κB in a model of peripheral axonal degeneration [40]. Yet, the effect of EPO on GLAST-expression upon simultaneous inhibition of NF-κB remains to be further investigated.

Interestingly, EPO protection of cultured neurons against glutamate neurotoxicity can be blocked by EGTA [26] suggesting a critical role for trace metals, in particular zinc, in resistance of cells to glutamate toxicity. Furthermore, it was shown that hippocampal perfusion with Ca-EDTA, a membrane-impermeable zinc chelator [41], increased the concentration of extracellular Glu. The hippocampal synaptic neurotransmission and synaptic plasticity is thought to be modulated by crosstalk between zinc which is co-released with Glu and calcium through calcium channels. These studies increase the physiological value of EPO-induced up-regulation of MT [22] which acts not only as free radical scavenger but also plays a pivotal role in cellular distribution of zinc [42].

Aging of the cell is featured by damage to the cell membranes and mitochondria resulting in LDH leakage and increased concentrations of intracellular lactate [35]. Among many other factors accelerating the development of these hallmarks of neural cell aging not the least place is given to accumulation of free radicals and glutamate [35]. Thus, exposure of rat neurons [43], C6 glioma cells and cerebral endothelial cells [44,45] to 1.0 mM glutamate increased the formation of reactive oxygen species, including superoxide radicals, and induced caspase-3 activation, DNA fragmentation, cell detachment [45] and mitochondrial dysfunction illustrated by impairment of oxygen consumption, glutathione depletion [43,44] three-fold increase of oxygen radicals [44]. Both glutathion and MT are known to be induced by EPO [34,22]. Our study shows that until 14 day in culture astroglial cells show high resistance to glutamate- and hypoxia-induced toxicity evidenced by relatively low leakage of LDH into the culture medium.

Aging of astrocytes in culture by itself, without any additional factors led to 5 fold increase of LDH leakage (250U/ml). The decreased resistance of aged astrocytes against environmental stresses such as glutamate or hypoxia is illustrated by further dramatic increase of LDH release up to 7-8 fold compared to similarly treated young astrocytes. Our results are consistent with other reports demonstrating glutamate-induced mitochondrial depolarisation, a significantly higher release of LDH and formation of ROS in cortical slices of aged rats compared to that of young rats [46]. Treatment with EPO decreased the LDH leakage in all cell age groups in concentration dependent manner. In aged astrocytes administration of EPO minimized the LDH release from astrocytes more than twice, most likely via activation of ROS scavenging systems such as glutathion [43,44,34] and MT [22,23] known to be decreased in aged cells.

As demonstrated here silencing of EPOR led to increased apoptosis astroglial cells, with or without exposure to Glu. No difference was observed between 1 and 5 mM Glu on cell apoptosis. This finding correlated with the previous study that demonstrated the mean percent cell death at glutamate concentrations between 1 to 20 mM was approximately equal [47]. The present findings showing the crucial role of EPOR in survival of astrocytes is in accordance with other reports demonstrating severe embryonic neurogenesis defects in animals null for either the EPO or EPOR gene, suggesting that EPOR is essential for EPO action during embryonic neurogenesis [48]. The impairment of glutamate uptake in EPOR-siRNA transfected APC hints to the possible decreased activity of glutamate transporters. The association between EPO-EPOR signalling pathway and GS is seen from slightly diminished expression of GS protein detected in EPOR-siRNA transfected APC by Western blotting and dramatically decreased the GS-activity both under NC and HC.

Hypoxia and aging in culture conditions lead to increased expression of EPOR (Figure 8) suggesting EPOR as a marker of cell vulnerability. This assumption is further supported by our in vivo data showing a prominent increase in EPOR expression during aging in the brains of wild type and APP/PS1 mice (Figure 10). Here we also show decreased EPO expression in APP/PS1 in comparison with age-matched WT controls. This finding hints at the limited capacity of brain cells to up-regulate EPO as a mechanism of defence against hypoxic and Glu-induced cytotoxicity during Alzheimer’s-like pathologies. The scenario of EPOR upregulation as a self-defensive mechanism under Alzheimer’s-like neurodegeneration is supported by several studies showing a protective effects of EPO against Amyloid beta (Aβ) toxicity. EPO prevents early and late apoptotic neuronal injury during Aβ toxicity involving the EPOR signalling and nuclear translocation of NFκB [49]. An in vivo study of Arabpoor et al. has shown a proliferative effect of EPO on neurons in the dentate gyrus of rats with streptozotocin-induced AD-like defects [50]. The clinical relevance of our data showing a prominent increase in overall expression of EPOR in the brains of APP/PS1 mice is reflected by the studies showing increased EPOR in hippocampal and cortical astrocytes in patients with mild cognitive impairment and sporadic AD [51]. Human study of Brettschneider et al. showed that EPO concentration in the cerebrospinal fluid of AD patients did not differ from their age-matched controls [52]. This fact was explained by the existence of either a relative deficiency of EPO in the brain of AD patients and/or by the removal of free EPO molecules from brain intercellular fluid by increased numbers of EPOR. Our data support this assumption of Brettschneider et al. [52] by demonstration of decreased EPO expression in the brains of transgenic APP/PS1 mice which correlates with increased EPOR expression when compared with their age-matched WT controls.

In conclusion, the present study shows for the first time the direct correlation between the extent of culture-induced aging (differentiation) of astrocytes and the efficacy of EPO to improve the extracellular glutamate clearance and metabolism. Our results demonstrate the synchronizing effects of EPO on the individual chains of glutamate turnover and detoxification in young and aged astroglial cells. On one hand administration of EPO activates the glutamate transporters that reduces the extracellular concentrations of glutamate and on the other hand, increases the enzymatic activity of GS that contributes to catalysis of intracellular glutamate. The protective effects of EPO shown here depend on functionality of EPOR, without which astrocytes undergo overall apoptosis. These effects of EPO on glutamate turnover allow to consider EPO as a potent neuroprotective agent for anti-aging interventions both during normal aging and age-related degenerative diseases. Based on our in vivo data showing increased EPOR and decreased EPO in a transgenic mouse model of AD in comparison with their age-matched WT controls and increase in EPO and EPOR induced by aging in both normal and AD mice it can be suggested that EPO and EPOR can serve as possible markers of brain cell vulnerability during aging and Alzheimer’s pathology. Thus, the correlation between EPO/EPOR expression and markers of glial glutamate uptake and metabolism during AD pathology will be addressed in future studies.
